# Role of recombinant human granulocyte colony-stimulating factor in development of cancer-associated venous thromboembolism in lung cancer patients who undergo chemotherapy

**DOI:** 10.3389/fimmu.2024.1386071

**Published:** 2024-05-31

**Authors:** Yi Cheng, Yunfeng Zhao, Mei Xu, He Du, Jinyuan Sun, Qihuan Yao, Jianmin Qu, Song Liu, Xuejun Guo, Wei Xiong

**Affiliations:** ^1^ Department of Pulmonary and Critical Care Medicine, Xinhua Hospital, Shanghai Jiaotong University School of Medicine, Shanghai, China; ^2^ Department of Pulmonary and Critical Care Medicine, Punan Hospital, Shanghai, China; ^3^ Department of General Practice, North Bund Community Health Service Center, Shanghai, China; ^4^ Department of Oncology, Shanghai Pulmonary Hospital, Tongji University School of Medicine, Shanghai, China; ^5^ Department of Traditional Chinese Medicine, Kongjiang Hospital, Shanghai, China; ^6^ Department of Intensive Care, Tongxiang First People’s Hospital, Tongxiang, China; ^7^ Department of Cardiovascular Medicine, Graduate School of Medicine, Kyoto University, Kyoto, Japan

**Keywords:** venous thromboembolism, chemotherapy, granulocyte colony-stimulating factor, lung cancer, rhG-CSF

## Abstract

**Background:**

The role of recombinant human granulocyte colony-stimulating factor (rhG-CSF), especially the long-acting factor in the development of cancer-associated venous thromboembolism (VTE) in lung cancer patients who undergo chemotherapy has been understudied, although the use of rhG-CSF has been reported to be associated with an increased risk of VTE.

**Methods:**

We retrospectively reviewed 1,673 lung cancer patients who underwent hospitalized chemotherapy. We performed propensity score matching to offset confounding factors related to cancer-associated VTE development and classified the patients into short-acting (N = 273), long-acting (N = 273), and no rhG-CSF (N = 273) groups. The primary outcome was cumulative cancer-associated VTE development three months after all cycles of chemotherapy.

**Results:**

The overall VTE incidence in the short-acting, long-acting, and no rhG-CSF groups was 5.5%, 10.3%, and 2.2%, respectively (P <0.001). The VTE incidence in the long-acting rhG-CSF group was higher than that in the short-acting (P = 0.039) and no rhG-CSF groups (P <0.001). The VTE incidence in the short-acting rhG-CSF group was higher than that in the no rhG-CSF group (P = 0.045). The use of rhG-CSF (hazard ratio [HR] 2.337; 95% confidence interval [CI] [1.236–5.251], P = 0.006) was positively correlated with VTE development among all patients, whereas the use of long-acting rhG-CSF (HR 1.917, 95% CI [1.138–4.359]; P = 0.016), was positively correlated with VTE development in patients receiving rhG-CSF.

**Conclusion:**

The use of rhG-CSF, especially long-acting rhG-CSF, increases the risk of cancer-associated VTE development compared to no rhG-CSF use in lung cancer patients who undergo hospitalized chemotherapy.

## Introduction

Lung cancer is the second most common type of cancer with the highest mortality rate globally (1, 2). Venous thromboembolism (VTE), usually defined as pulmonary embolism (PE), deep venous thrombosis (DVT), or a combination of both (3), is a common and life-threatening condition in patients with cancer, including lung cancer (4–7). Immediately following pancreatic and gastric cancers, which carry a very high risk of VTE, lung cancer is one of the cancer types that carries a high risk of VTE development and recurrence (5, 6) and is associated with an increased mortality rate (8–11).

Elevated levels of leukocytes, platelets, and tissue factor-positive microvesicles ((TF^+^ MVs) are major predisposing factors that, alone or in combination, increase the risk of cancer-associated VTE. Neutrophilia plays an important role in the development of VTE in patients with lung cancer. Tumor-derived granulocyte colony-stimulating factor (G-CSF) results in the elevation of neutrophil levels that release neutrophil extracellular traps (NETs), which increases the risk of VTE in patients with lung cancer (12). In a prospective observational study, 33 (14.5%) of 227 patients with lung cancer were diagnosed with tumor-related leukocytosis. Among these 33 patients, 16 demonstrated high serum G-CSF levels (13). G-CSF is a cytokine that supports survival and stimulates the proliferation of neutrophil progenitors, promotes their differentiation into mature neutrophils, causes premature release of neutrophils from the bone marrow, enhances phagocytic capacity, generates superoxide anions, and kills bacteria. It is often associated with leukocytosis and neutrophilia and is also produced by various tumors and cancer cells (14–16).

Neutropenia especially febrile neutropenia (FN) is a life-threatening complication of chemotherapy for patients with cancer including lung cancer. It can cause severe infections that significantly increase the mortality rates. The most vital prognostic factor for cancer patients with FN is the recovery of neutrophil count. Recombinant human granulocyte colony-stimulating factor (rhG-CSF) is a hematopoietic growth factor that promotes the proliferation and differentiation of neutrophils (15, 17). It has been demonstrated to increase neutrophil counts to alleviate the magnitude of chemotherapy-induced FN in patients with lung cancer (17). In recent years, mecapegfilgrastim, a long-acting pegylated rhG-CSF, has become an effective and well-tolerated regimen for the stewardship of chemotherapy-induced FN in cancer patients who undergo myelosuppressive chemotherapy in China (18). Therapeutic equivalence exists between short-acting and long-acting rhG-CSF agents (19, 20). Long-acting rhG-CSF has the advantage of being more user-friendly, with the disadvantage of higher cost, compared to short-acting rhG-CSF (19).

As an increase in tumor-derived G-CSF levels can induce the development of VTE, the administration of exogenous rhG-CSF may have a certain effect on VTE development in patients with lung cancer who undergo chemotherapy. Nevertheless, the role of rhG-CSF in cancer-associated VTE development in cancer patients is inconsistent (21, 22). Furthermore, studies on the role of rhG-CSF in cancer-associated VTE development specific to lung cancer patients undergoing chemotherapy are lacking. In addition, the role of short-acting and long-acting rhG-CSF in VTE development in lung cancer patients undergoing chemotherapy has been understudied. Therefore, this study was conducted to address this issue.

## Methods

### Study design

A retrospective study was conducted to determine the role of rhG-CSF use including short- and long-acting rhG-CSF, in the development of cancer-associated VTE in lung cancer patients who underwent chemotherapy. Patients with primary lung cancer who had undergone hospitalized chemotherapy were reviewed. According to the recommendations of the American Society of Clinical Oncology (ASCO) guidelines on the use of hematopoietic colony-stimulating factors (20), rhG-CSF had been applied to lung cancer patients during chemotherapy under the following circumstances: (1) primary prophylaxis against chemotherapy-induced FN with rhG-CSF was adopted in patients who had an approximately 20% or higher risk for FN based on the risk factors for FN development, mainly including age >65 years and/or other comorbidities; (2) secondary prophylaxis against chemotherapy-induced FN with rhG-CSF was adopted for patients who experienced a neutropenic complication for which primary prophylaxis was not received from a prior cycle of chemotherapy; and (3) rhG-CSF treatment was adopted in patients with established FN who were at high risk of infection or were prone to have poor clinical outcomes. FN was defined as an oral temperature higher than 38.3°C or two consecutive readings higher than 38.0°C for 2 h and an absolute neutrophil count (ANC) being less than 0.5 × 10^9^/L or expected to fall below 0.5 × 10^9^/L (23). As per the recommendations in the guidelines, the short-acting rhG-CSF, which was filgrastim, was administered at approximately 5 μg/kg subcutaneously once daily until ANC reached 2 to 3 × 10^9^/L, whereas the long-acting rhG-CSF, mecapegfilgrastim, was administered 6 mg once subcutaneously 24 h–72 h after the last day of chemotherapy. The option of long-acting or short-acting rhG-CSF agents was up to attending physicians depending on convenience, cost, and clinical situation, as there are no specific guidelines on how to choose to use them. Antibiotic therapy has been used in patients with established FN (20, 24). Patients usually undergo routine blood tests twice a week after chemotherapy.

In accordance with the recommendations in the guidelines (6), the risk of VTE was assessed periodically for all lung cancer patients who had undergone hospitalized chemotherapy. During chemotherapy or follow-up, for patients with a gestalt of clinical VTE, a positive pretest prediction score and an abnormal age-adjusted D-dimer level (3), computed tomography pulmonary angiography (CTPA), compression ultrasonography (CUS) of the lower and upper extremities, and/or planar ventilation/perfusion (V/Q) scan were performed to determine the presence or absence of VTE (3, 5–7). Meanwhile, thromboprophylaxis with low molecular weight heparin was administrated during hospitalization, unless there were contraindications to thromboprophylaxis (5, 6, 25).

According to the type of rhG-CSF used throughout all cycles of chemotherapy, patients were classified into three groups: the short-acting rhG-CSF group, in which patients had received isolated short-acting rhG-CSF; the long-acting rhG-CSF group, in which patients had received isolated long-acting rhG-CSF; and the no rhG-CSF group, in which patients had received no rhG-CSF. The use of rhG-CSF was defined as patients receiving rhG-CSF in at least one cycle of chemotherapy, whereas no use of rhG-CSF was defined as patients receiving no rhG-CSF throughout all cycles of chemotherapy.

The baseline was defined as the initiation of the first cycle of chemotherapy, whereas the endpoint was defined as the return visit three months after all cycles of chemotherapy. The primary outcome was the cumulative incidence of VTE development, whereas the secondary outcome was the growth multiple of ANC. Data on VTE development and death were collected every month. Growth multiple of ANC was defined as the mean multiple of ANC after rhG-CSF use at the beginning of the next chemotherapy to that before rhG-CSF use at the end of previous chemotherapy for the short-acting and long-acting rhG-CSF groups and mean multiple of the ANC at the beginning of the next chemotherapy to that at the end of previous chemotherapy for the no rhG-CSF group. The overall cumulative VTE incidence were compared among the three groups. Time-dependent cumulative VTE incidence from baseline to endpoint was compared among the three groups. The correlation between VTE development and rhG-CSF use was also analyzed. Correlations among VTE development, rhG-CSF dose, and ANC growth multiple were analyzed.

The data required for the study were obtained using the existing electronic medical record system. Patients and the public were not involved in the design, conduct, reporting, or dissemination plans of our research. All the authors vouched for the completeness, fidelity, and accuracy of the data. All the authors contributed to the writing of the manuscript. All the authors have read and approved the submitted version of the manuscript for publication. The study protocol was approved by the Institutional Review Board of Shanghai Xinhua Hospital (XHEC-QT-2021–056).

### Study population

Eligible patients were included in the present study based on the inclusion and exclusion criterion. The inclusion criteria were as follows: (1) all eligible patients were 18 years or older, (2) all eligible patients had an objectively established histopathological diagnosis of primary lung cancer, and (3) all eligible patients underwent standard hospitalized chemotherapy with or without rhG-CSF use and were followed up for at least 3 months. The exclusion criteria were as follows: (1) patients who had other known primary cancers other than lung cancer, (2) patients with a history of chronic thromboembolic disease(CTED) (3), and (3) patients who developed VTE prior to rhG-CSF use in those who underwent rhG-CSF therapy.

### Statistical analysis

Propensity score matching was adopted to offset the bias of potential confounding factors that are highly associated with cancer-associated VTE. Based on previous literature (5, 6, 25), we matched previous VTE, familial and/or acquired hypercoagulability due to reasons other than cancer, medical comorbidities (infection, renal disease, pulmonary disease, congestive heart failure, or arterial thromboembolism), performance status, metastasis, VTE-related anticancer therapies (major surgery, immunotherapy, protein kinase inhibitors, hormonal or antiangiogenic therapies), rhG-CSF, hematopoietic growth factors other than rhG-CSF, central venous catheter (CVC), prolonged immobilization, D-dimer, Khorana score, thromboprophylaxis, and concomitant antiplatelet use among the three groups. In propensity score matching analysis, nearest-neighbor (greedy) matching without replacement was adopted (26). A 1:1:1 ratio within a caliper width of 0.2 (27) of the standard deviation of the logit of the propensity score was adopted for the number ratio of patients among the short-acting, long-acting, and no rhG-CSF groups, respectively. The matching algorithm first selected a patient in the no rhG-CSF group and then selected one patient in the other two groups who had a linear propensity score closest to that of the first selected patient.

Measurement data were presented as mean ± standard deviation or median with interquartile range, depending on whether they conformed to a normal distribution. Categorical data were presented as percentages. The comparison of measurement data was performed using T-test or ANOVA. The comparison of rates was performed using the chi-square test. The time-to-event cumulative incidence of VTE among the three groups was compared using the Kaplan–Meier method. The correlation between VTE development and risk factors, including rhG-CSF use in all patients and those receiving rhG-CSF, was analyzed using sequential univariable and multivariable Cox proportional hazard models, respectively. The covariates in the models were selected according to previous guidelines (5, 6, 25) and 10 events per variable rule of thumb (28). Pearson correlation analysis was adopted for the association between VTE development, rhG-CSF dose, and ANC growth multiple. Statistical analyses were performed using SPSS 26 and R software (version 3.6.1; R Project for Statistical Computing). Statistical significance was defined as a P-value being less than 0.05.

## Results

### Characteristics of patients

In line with the inclusion criteria, 1,673 eligible patients between January 2018 and December 2023 from all participating hospitals were included in the current study. In line with the exclusion criteria, 103 patients were excluded from this study. Thus, 1,570 patients were recruited based on the inclusion and exclusion criteria. After propensity score matching, 819 patients were included in the final analysis set. The numbers of patients in the short-acting, long-acting, and no rhG-CSF groups were 273, 273, and 273, respectively. The mean age of the patients was 69.4 years old. Among them, 351 patients were female and 468 patients were male. All VTE-related variables were similar among the three groups (**Table 1**).

**Table 1:** Characteristics of patients.

	Short-acting rhG-CSF (N = 273)	Long-acting rhG-CSF (N = 273)	No rhG-CSF (N = 273)	P-value
Age-years	68.6 (59.2–77.6)	70.0 (62.5–80.2)	69.5 (58.1–78.8)	0.137
Sex(female)-no.(%)	110 (40.3)	123 (45.1)	118 (43.2)	0.526
BMI-kg/m^2^	25.5 (18.4–28.3)	22.4 (19.7–26.2)	26.7 (20.1–31.4)	0.231
Smoker-no. (%)	158 (57.9)	145 (53.1)	162 (59.3)	0.308
Histopathology-no.(%) Adenocarcinoma Non-adenocarcinoma	163 (59.7) 110 (40.3)	151 (55.3) 122 (44.7)	177 (64.8) 96 (35.2)	0.076
TNM stage Stage I Stage II Stage III Stage IV	10 (3.7) 27 (9.9) 66 (24.2) 170 (62.3)	8 (2.9) 24 (8.8) 73 (26.7) 168 (61.5)	14 (5.1) 30 (11.0) 56 (20.5) 173 (63.4)	0.578
Metastasis-no.(%)	170 (62.3)	168 (61.5)	173 (63.4)	0.906
PS score-points	1.6 ± 0.8	1.5 ± 1.0	1.8 ± 0.9	0.831
Chemotherapy cycles-times	5.5 ± 2.0	5.6 ± 2.3	5.1 ± 2.1	0.726
Chemotherapy regimens-no.(%) Docetaxel + platinum Etoposide + platinum Gemcitabine + platinum Pemetrexed + platinum Paclitaxel + platinum Others	41 (15.0) 40 (14.7) 35 (12.8) 50 (18.3) 38 (13.9) 69 (25.3)	47 (17.2) 45 (16.5) 38 (13.9) 44 (16.1) 35 (12.8) 64 (23.4)	51 (18.7) 33 (12.1) 30 (11.0) 65 (23.8) 31 (11.4) 63 (23.1)	0.501
Platinum-based chemotherapy- no.(%)	218 (79.9)	222 (81.3)	231 (84.6)	0.334
RECIST-no.(%) CR PR SD PD	39 (14.3) 70 (25.6) 90 (33.0) 74 (27.1)	45 (16.5) 66 (24.2) 97 (35.5) 65 (23.8)	33 (12.1) 50 (18.3) 101 (37.0) 89 (32.6)	0.126
Fever-no.(%)	109 (39.9)	123 (45.1)	28 (10.3)	<0.001
ANC-×10^9^/L Before rhG-CSF After rhG-CSF	0.5 (0.3–1.0) 2.6 (1.2–4.4)	0.4 (0.2–0.9) 5.6 (3.3–8.0)	3.8 (1.5–6.2) 5.8 (3.7–7.9)	<0.001 0.089
rhG-CSF Prophylaxis-no.(%) Treatment-no.(%)	83 (30.4) 190 (69.6)	108 (39.6) 165 (30.4)		0.025
Total cumulative dose of rhG-CSF-mg	16.2 ± 6.9	17.5 ± 6.4		0.838
Previous VTE-no.(%)	10 (3.7)	13 (4.8)	15 (5.5)	0.592
Hypercoagulability-no. (%)	27 (9.9)	25 (9.2)	28 (10.3)	0.908
Medical comorbidities-no. (%)	138 (50.5)	136 (49.8)	134 (49.1)	0.943
Central venous catheter-no. (%)	106 (38.8)	103 (37.7)	109 (39.9)	0.870
Other hematopoietic growth factors-no.(%)	126 (46.2)	120 (44.0)	130 (47.6)	0.688
Other anticancer therapies-no.(%)	213 (78.0)	211 (77.3)	217 (79.5)	0.818
Prolonged immobilization-no.(%)	163 (59.7)	160 (58.6)	168 (61.5)	0.779
D-dimer-mg/L	2.4 ± 1.2	2.3 ± 1.3	2.5 ± 1.3	0.886
Khorana score-points	1.5 ± 1.0	1.3 ± 0.7	1.4 ± 0.7	0.237
Thromboprophylaxis-no.(%)	233 (85.3)	235 (86.1)	230 (84.2)	0.832
Concomitant antiplatelet use-no.(%)	50 (18.3)	55 (20.1)	58 (21.2)	0.687

Hypercoagulability was defined as familial and/or acquired hypercoagulability for reasons other than cancer. Medical comorbidities were defined as at least one of infection, renal disease, pulmonary disease, congestive heart failure, or arterial thromboembolism. Other hematopoietic growth factors were defined as erythropoietin or thrombopoietin. Other anticancer therapies included major surgery, immunotherapy, protein kinase inhibitors, and hormonal or antiangiogenic therapies.

rhG-CSF, recombinant human granulocyte colony-stimulating factor; no., number; BMI, body mass index; TNM, tumor-node-metastasis; PS, performance status; RECIST, response evaluation criteria in solid tumors; CR, complete response; PR, partial response; SD, stable disease; PD, progressive disease; ANC, absolute neutrophil count; CVC, central venous catheter.

### Comparison of overall VTE incidence and ANC growth multiple among three groups

**Figure 1:**
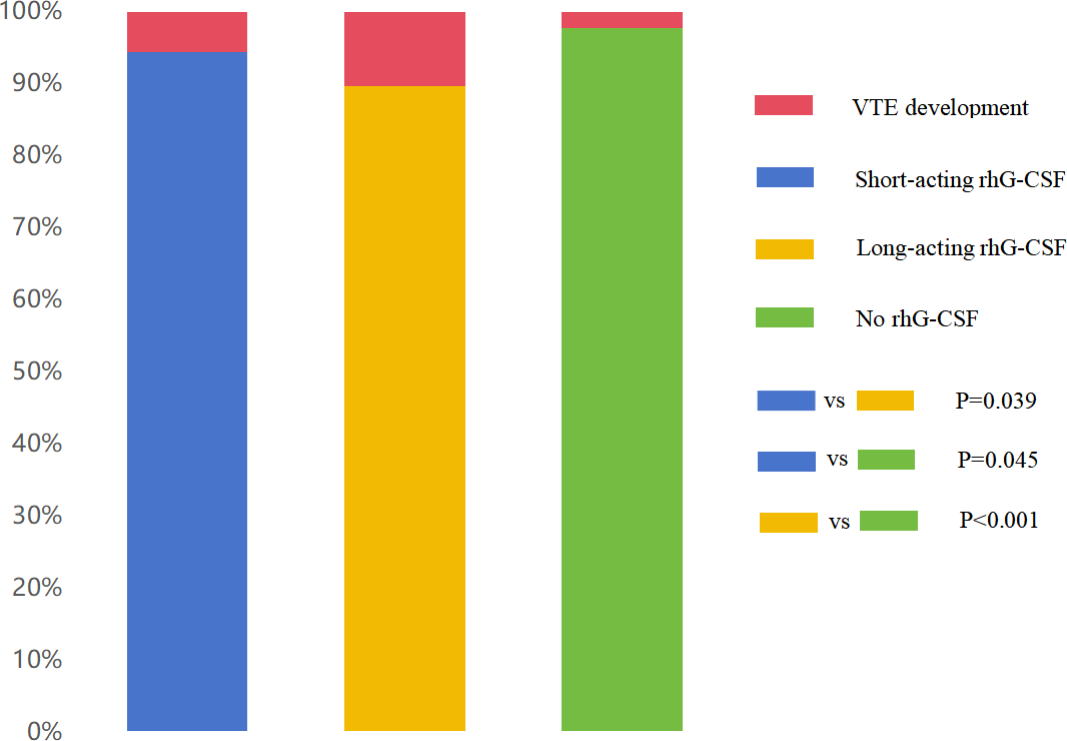
Comparison of overall VTE incidence among three groups. The VTE incidence in the long-acting rhG-CSF group (10.3%) was higher than that in the short-acting rhG-CSF group (5.5%) (P = 0.039) and no rhG-CSF group (2.2%) (P <0.001). The VTE incidence in the short-acting rhG-CSF group was also higher than that in the no rhG-CSF group (P = 0.045). VTE, venous thromboembolism; rhG-CSF, recombinant human granulocyte colony-stimulating factor.

All 819 patients underwent VTE imaging tests at least once from the baseline to the endpoint. The median times of VTE imaging tests were 3.6 (95% confidence interval [CI][1.2–6.0]), 3.2 (95% CI[1.5–5.1]), 3.9 (95% CI[2.2–5.6]) times in the short-acting, long-acting, and no rhG-CSF groups, respectively. (P = 0.005) The overall VTE incidence were 15 (5.5%), 28 (10.3%), and 6 (2.2%) in the short-acting, long-acting, and no rhG-CSF groups, respectively (P <0.001). The VTE incidence was higher in patients who underwent rhG-CSF therapy than in those who did not (7.9% vs 2.2%) (P = 0.001). The numbers of patients with PE, DVT, and PE combined with DVT were (5, 5, 5), (10, 10, 8), and (3, 2, 1) in the short-acting, long-acting, and no rhG-CSF groups, respectively (P = 0.941). The pairwise comparison demonstrated that the VTE incidence in the long-acting rhG-CSF group was higher than that in the short-acting rhG-CSF group (P = 0.039) and the no rhG-CSF group (P <0.001). The VTE incidence in the short-acting rhG-CSF group was also higher than that in the non-rhG-CSF group (P = 0.045) (**Figure 1**).

The growth multiple of ANC was 5.2 (95% CI[3.5–9.1]), 14.2 (95% CI[10.1–18.3]), and 1.5 (95% CI[0.5–2.8]) folds in the short-acting, long-acting, and no rhG-CSF groups, respectively (P <0.001). The growth multiples of ANC in the long-acting rhG-CSF group were higher than those in the short-acting rhG-CSF group (P = 0.001) and no rhG-CSF groups (P <0.001), respectively. The growth multiple of ANC in the short-acting rhG-CSF was more than that in the no rhG-CSF group (P = 0.001).

### Comparison of time-dependent cumulative VTE incidence among three groups

**Figure 2:**
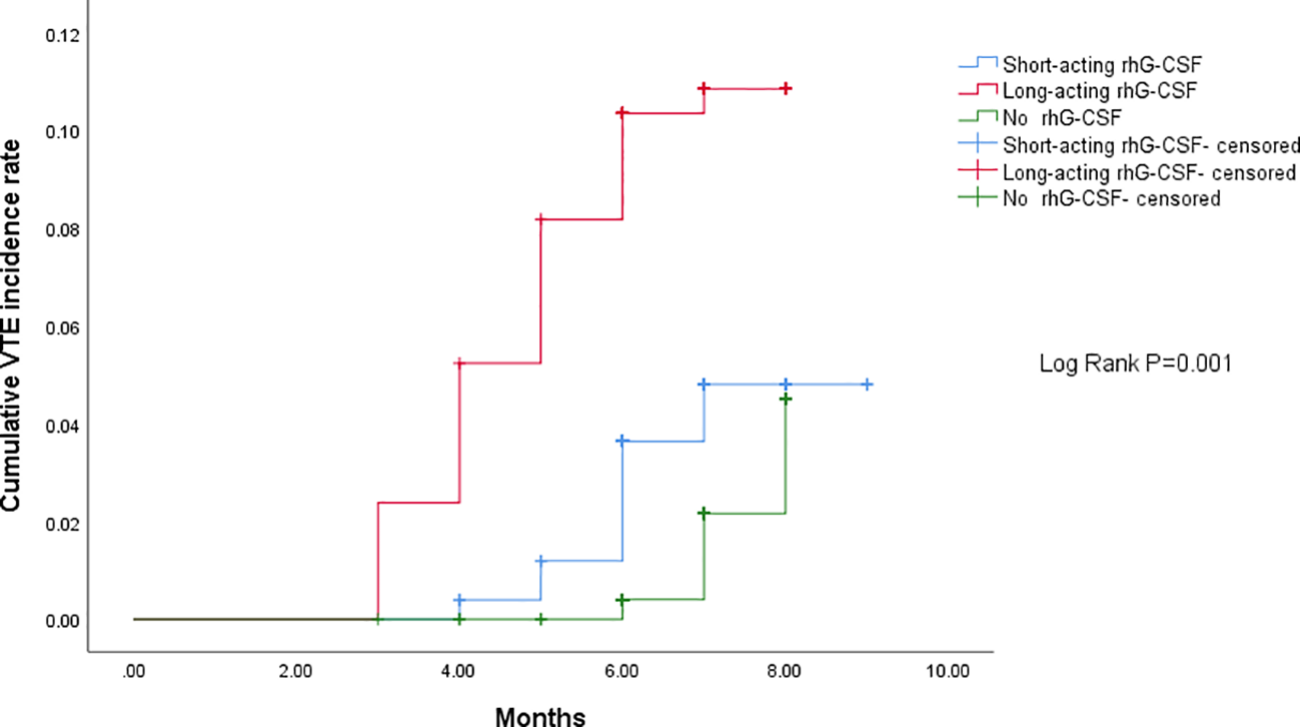
Comparison of time-dependent cumulative VTE incidence among three groups. The patients in the long-acting rhG-CSF group developed VTE sooner than those in the short-acting rhG-CSF group, who developed VTE sooner than those in the no rhG-CSF group (P = 0.001). The pairwise P- values of time-dependent cumulative VTE incidence for long-acting rhG-CSF vs short-acting rhG-CSF, long-acting rhG-CSF vs no rhG-CSF, and short-acting rhG-CSF vs no rhG-CSF were 0.030, <0.001, and 0.035, respectively. VTE, venous thromboembolism; rhG-CSF, recombinant human granulocyte colony-stimulating factor.

The median time from baseline to VTE development were 5.5 (95% CI[3.1–7.6]), 3.4 (95% CI[1.8–5.0]), and 6.6 (95% CI[4.0–9.2]) months in the short-acting, long-acting, and no rhG-CSF groups, respectively (P = 0.024). The median chemotherapy cycles from baseline to VTE development were 5.4 (95% CI[3.1–8.3]), 3.3 (95% CI[2.2–7.4]), and 6.2 (95% CI[3.2–8.9]) cycles in the short-acting, long-acting, and no rhG-CSF groups, respectively (P = 0.117). The median time from the initiation of rhG-CSF use to VTE development was 3.8 (95% CI[1.5–6.1]) and 2.3 (95% CI[1.4–3.9]) months in the short-acting and long-acting rhG-CSF groups, respectively (P = 0.001). The median dose of rhG-CSF used before VTE development was 12.3 (95% CI[7.1–15.1])mg and 7.7 (95% CI[5.1–11.8])mg in the short-acting and long-acting rhG-CSF groups, respectively (P <0.001). The patients in the long-acting rhG-CSF group developed VTE sooner than those in the short-acting rhG-CSF group, who developed VTE sooner than those in the no rhG-CSF group (P = 0.001). The pairwise P-values of time-dependent cumulative VTE incidence of long-acting rhG-CSF vs short-acting rhG-CSF, long-acting rhG-CSF vs no rhG-CSF, and short-acting rhG-CSF vs no rhG-CSF were 0.030, <0.001, and 0.035, respectively (**Figure 2**).

### Correlation between VTE development and risk factors

As there were 49 events of VTE development, according to the 10 events per variable rule of thumb (28), we incorporated four covariates into the Cox regression models: Khorana score (≥c vs <2), PS score (≥c vs <2), metastasis (yes vs no), and previous VTE history (yes vs no).

Multivariable Cox regression analysis for all patients demonstrated that the use of rhG-CSF (hazard ratio [HR] 2.337, 95% CI [1.236–5.251], P = 0.006), metastasis (HR 4.370, 95% CI [1.836–10.403], P = 0.001), and previous VTE history (HR 2.280, 95% CI [1.446–6.906], P = 0.021) were positively correlated with the VTE development finally (**Table 2**). Multivariable Cox regression analysis for patients receiving rhG-CSF demonstrated that the use of long-acting rhG-CSF (HR 1.917, 95% CI [1.138–4.359], P = 0.016), PS ≥2 (HR 1.723, 95% CI [1.040–2.856], P = 0.035), and metastasis (HR 4.510, 95% CI [1.999–10.173], P <0.001) were positively correlated with the VTE development finally (**Table 3**).

**Table 2:** Correlation between VTE development and risk factors in all patients.

	Hazard Ratio (95%CI) (univariable)	P value	Hazard Ratio (95%CI) (multivariable)	P-value
rhG-CSF vs Non rhG-CSF	2.813 (1.135–5.573)	0.001	2.337 (1.236–5.251)	0.006
Khorana score ≥2 vs Khorana score <2	1.236 (0.534–2.863)	0.620		
PS ≥2 vs PS <2	2.754 (1.076–7.051)	0.035	1.504 (0.698–3.241)	0.298
Metastasis vs No metastasis	4.989 (2.145–11.603)	<0.001	4.370 (1.836–10.403)	0.001
Previous VTE vs No previous VTE	2.395 (1.560–10.250)	0.009	2.280 (1.446–6.906)	0.021

VTE, venous thromboembolism; CI, confidence interval; rhG-CSF, recombinant human granulocyte colony-stimulating factor; PS, performance status.

**Table 3:** Correlation between VTE development and risk factors in patients receiving rhG-CSF.

	Hazard Ratio (95%CI) (univariable)	P-value	Hazard Ratio (95%CI) (multivariable)	P-value
Long-acting vs Short-acting	2.286 (1.174–5.015)	0.001	1.917 (1.138–4.359)	0.016
Khorana score ≥2 vs Khorana score <2	1.146 (0.515–2.553)	0.739		
PS ≥2 vs PS <2	2.092 (1.280–3.419)	0.003	1.723 (1.040–2.856)	0.035
Metastasis vs No metastasis	5.458 (2.470–12.061)	<0.001	4.510 (1.999–10.173)	<0.001
Previous VTE vs No previous VTE	1.436 (0.338–6.090)	0.624		

VTE, venous thromboembolism; CI, confidence interval; rhG-CSF, recombinant human granulocyte colony-stimulating factor; PS, performance status.

### Correlation among VTE development, rhG-CSF dose, and ANC growth multiple

Pearson correlation analysis indicated that the dose of short-acting (R = 0.509, P <0.001) and long-acting rhG-CSF (R = 0.833, P <0.001) were positively correlated with ANC growth multiple in the short-acting and long-acting rhG-CSF groups, respectively. ANC growth multiple in the short-acting (R = 0.437, P <0.001) and long-acting rhG-CSF (R = 0.534, P <0.001) groups was positively correlated with VTE development in the short-acting and long-acting rhG-CSF groups, respectively. Finally, the dose of short-acting (R = 0.463, P <0.001) and long-acting rhG-CSF (R = 0.573, P <0.001) were found positively correlated with the VTE development in the short-acting and long-acting rhG-CSF groups, respectively.

## Discussion

The major findings derived from the current results are as follows: (1) For lung cancer patients being followed up for 3 months after all cycles of chemotherapy, rhG-CSF use yielded more cancer-associated VTE than no rhG-CSF use, whereas long-acting rhG-CSF use yielded more cancer-associated VTE than short-acting rhG-CSF use. (2) rhG-CSF especially long-acting rhG-CSF, is an independent risk factor for VTE development. To the best of our knowledge, this is the first study to explore the role of rhG-CSF in cancer-associated VTE development specific for isolated lung cancer patients. In addition, the role of short-acting and long-acting rhG-CSF in VTE development for lung cancer patients undergoing chemotherapy was explored for the first time.

Neutrophilia is the major mechanism in the process of cancer-associated VTE development in patients with lung cancer (12), while rhG-CSF can cause a proliferation of granulocytes (15, 17). Theoretically speaking, rhG-CSF is a risk factor for VTE development in lung cancer patients receiving chemotherapy who are often in need of rhG-CSF treatment or prophylaxis. In addition, in a randomized controlled trial, subjects receiving 5 μg/kg filgrastim subcutaneously for 4 days had significantly enhanced platelet aggregation induced by clinically relevant platelet agonists, including adenosine diphosphate, collagen, and arachidonic acid, compared to those receiving placebo. Enhanced platelet aggregation translates to a 75% increase in platelet activation (29). Platelet aggregation and activation induced by G-CSF may also put lung cancer patients at a risk of cancer-associated VTE.

In a prospective observational study by Khorana et al., the risk factors for chemotherapy-associated VTE were analyzed among 3,003 cancer patients, including 574 (19.1%) lung cancer patients. In the multivariate analysis, patients with cancer of the upper gastrointestinal tract, lung, and lymphoma who received white-cell growth factor use had a significantly increased risk of VTE (5.9%), than those without growth factor use (1.52%). (P <0.0001) (21). Since then, a series of studies have demonstrated that the application of G-CSF is associated with an increased risk of VTE in patients with gastroesophageal (30), colorectal (31), breast (32), and comprehensive cancers (33).

However, in a meta-analysis that evaluated the safety and efficacy of adding G-CSF to antibiotics when treating chemotherapy-induced FN in patients with cancer, no significant difference was found in the incidence of deep VTE (risk ratio 1.68, 95% CI [0.72–3.93], P = 0.23) in individuals treated with G-CSF plus antibiotics compared with those treated with antibiotics alone. Nevertheless, this result, derived from four randomized controlled trials including 389 participants, is low-quality evidence (22).

The results of the present study were in favor of the perspective that rhG-CSF use is associated with an increased risk of VTE in patients with lung cancer, compared with the absence of rhG-CSF. The use of rhG-CSF in combination with chemotherapy resulted in a higher VTE risk than isolated chemotherapy alone. Moreover, the present study further indicated that lung cancer patients who received long-acting rhG-CSF were more likely to develop VTE than those who received short-acting rhG-CSF. Owing to the better therapeutic efficacy of long-acting rhG-CSF in leukocyte boosting than short-acting rhG-CSF (34), long-acting rhG-CSF may correspondingly yield a higher risk of thrombosis due to its greater capacity for the elevation of leukocytes and longer drug maintenance effect.

The clinical implications of the current study are as follows: (1) Compared with lung cancer patients who do not undergo the rhG-CSF regimen, the risk of VTE development in those who undergo the rhG-CSF regimen should be more noteworthy. The use of rhG-CSF can be regarded as a cancer-associated VTE risk factor when assessing the VTE risk in this patient population. (2) Clinicians should pay more attention to the risk of cancer-associated VTE in patients receiving long-acting rhG-CSF than in those receiving short-acting rhG-CSF.

### Limitations

The limitations of this study must be acknowledged. First, although the adoption of propensity score matching in the current study may minimize bias caused by confounding factors, it is still inferior to randomized controlled trials. The retrospective nature of this study warrants further prospective investigation. Second, since the follow-up period may be too short to reflect the mortality rate, all-cause and VTE-related mortalities were not presented in the current study. Third, because the current study population included all hospitalized patients undergoing isolated chemotherapy, the results may not be applicable to ambulatory lung cancer patients undergoing chemotherapy. Fourth, there is a risk of a multiple comparison problem by comparing three groups, while adjusting for multiple comparisons could increase the chance of a Type-II error. Finally, considering that the follow-up period was 3 months after chemotherapy, the current results may not remain the same for a longer follow-up time. However, in previous studies, a 3-month follow-up period is safe enough to rule out VTE development (35, 36).

### Conclusions

In conclusion, the current study suggests that rhG-CSF, especially long-acting rhG-CSF, may lead to more VTE development than no rhG-CSF use in lung cancer patients who undergo hospitalized chemotherapy, which is an independent risk factor for cancer-associated VTE. These findings may be conducive to thromboprophylaxis in lung cancer patients undergoing chemotherapy.

## Data availability statement

The raw data supporting the conclusions of this article will be made available by the authors, without undue reservation.

## Ethics statement

The study protocol was approved by the institutional review boards of Shanghai Xinhua Hospital (XHEC-QT-2021–056). The studies were conducted in accordance with the local legislation and institutional requirements. The ethics committee/institutional review board waived the requirement for written informed consent for participation from the participants or the participants’ legal guardians/next of kin because (1) the study involves no more than minimal risk for patients, (2) the study cannot adversely affect the rights and welfare of patients, and (3) the study cannot be performed without the exemption of informed consent of patients.

## Author contributions

YC: Data curation, Formal analysis, Funding acquisition, Investigation, Methodology, Resources, Software, Validation, Visualization, Writing – original draft, Writing – review & editing. YZ: Formal analysis, Funding acquisition, Investigation, Methodology, Project administration, Resources, Supervision, Validation, Visualization, Writing – original draft, Writing – review & editing. MX: Formal analysis, Investigation, Methodology, Resources, Validation, Visualization, Writing – review & editing. HD: Data curation, Formal analysis, Investigation, Methodology, Resources, Software, Validation, Visualization, Writing – review & editing. JS: Data curation, Formal analysis, Investigation, Methodology, Validation, Visualization, Writing – review & editing. QY: Data curation, Formal analysis, Investigation, Methodology, Resources, Validation, Visualization, Writing – review & editing. JQ: Data curation, Formal analysis, Investigation, Resources, Software, Validation, Visualization, Writing – review & editing. SL: Data curation, Formal analysis, Methodology, Resources, Software, Validation, Visualization, Writing – review & editing. XG: Methodology, Project administration, Resources, Software, Supervision, Validation, Visualization, Writing – review & editing. WX: Visualization, Writing – original draft, Writing – review & editing, Conceptualization, Data curation, Formal analysis, Funding acquisition, Investigation, Methodology, Project administration, Resources, Software, Supervision, Validation.
